# Characteristics of coal resources in China and statistical analysis and preventive measures for coal mine accidents

**DOI:** 10.1007/s40789-023-00582-9

**Published:** 2023-04-18

**Authors:** Chaolin Zhang, Peizhong Wang, Enyuan Wang, Dapeng Chen, Chao Li

**Affiliations:** 1 Key Laboratory of Gas and Fire Control for Coal Mines, Ministry of Education, School of Safety Engineering, China University of Mining and Technology, Xuzhou, 221116 Jiangsu China; 2 State Key Laboratory of Coal Resources and Safe Mining, China University of Mining and Technology, Xuzhou, 221116 Jiangsu China; 3 Shaanxi Coal Chemical Industry Technology Research Institute Co., LTD, Xi’an, 710054 Shaanxi China

**Keywords:** Resource characteristics, Coal mine accidents, Security situation, Safety 4 + 4 model, Preventive measures

## Abstract

In the process of green and smart mine construction under the context of carbon neutrality, China's coal safety situation has been continuously improved in recent years. In order to recognize the development of coal production in China and prepare for future monitoring and prevention of safety incidents, this study mainly elaborated on the basic situation of coal resources and national mining accidents over the past five years  (2017–2021), from four dimensions (accident level, type, region, and time), and then proposed the preventive measures based on accident statistical laws. The results show that the storage of coal resources has obvious geographic characteristics, mainly concentrated in the Midwest, with coal resources in Shanxi and Shaanxi accounting for about 49.4%. The proportion of coal consumption has dropped from 70.2% to 56% between 2011 and 2021, but still accounts for more than half of the all. Meanwhile, the accident-prone areas are positively correlated with the amount of coal production. Among different levels of coal mine accidents, general accidents had the highest number of accidents and deaths, with 692 accidents and 783 deaths, accounting for 87.6% and 54.64% respectively. The frequency of roof, gas, and transportation accidents is relatively high, and the number of single fatalities caused by gas accidents is the largest, about 4.18. In terms of geographical distribution of accidents, the safety situation in Shanxi Province is the most severe. From the time distribution of coal mine accidents, the accidents mainly occurred in July and August, and rarely occurred in February and December. Finally, the "4 + 4" safety management model is proposed, combining the statistical results with coal production in China. Based on the existing health and safety management systems, the managements are divided into four sub-categories, and more specific measures are suggested.

## Introduction

Mining has been considered an inherently high-risk industry worldwide, and China is the world's largest producer and consumer of coal (Zhang et al. 2021a, b; Liu et al. 2022; Wang et al. 2020). According to the National Bureau of Statistics in 2021, the raw coal production of China was 4.13 billion tons, which has an increase of 5.7% over the previous year. The total annual energy consumption in China was 5.24 billion tons of standard coal, which has an increase of 5.2% over the previous year, of which coal consumption accounted for 56.0% of the total energy consumption (National Bureau of Statistics 2022). The "14th Five-Year Plan" released by the China government emphasizes the need to continue to strengthen the construction of green and intelligent mines and promote the energy consumption revolution (National Mine Safety Administration 2021). In this context, the coal safety situation in China showed a stable improvement and there is a continuous downward trend accidental deaths in coal mines. The overall safety situation in China's coal industry is expected to be further improved. There were altogether 790 coal accidents with 1433 fatalities over the past five years  (2017–2021), and the coal mine death rate per million tons decreased by 58% from 0.106 in 2017 to 0.044 in 2021. However, the coal mine accidents and related fatalities remain high across all industries due to many factors (Linghu et al. 2021; Qiu et al. 2022; Kong et al. 2022). Therefore, it is especially important to clarify the development trend of coal mine safety accidents, find the law of accident evolution, and make effective suggestions for the next step of accident prevention, precise prevention and control.

In recent years, many scholars have carried out a lot of research on coal mine safety accidents. Chen studied the development trend of coal mine accidents and analyzed the causes of accidents in terms of human factors (Chen et al. 2012). Mahdevari used the fuzzy TOPSIS model to evaluate downhole safety risk in underground coal mines (Mahdevari et al. 2014). Wang conducted statistical analysis on gas accidents in Chinese coal mines (Wang et al. 2014). Zheng investigated the coal dust explosion accidents and put forward the preventive countermeasures (Zheng et al. 2009). Yi expounded the unsafe behaviors and distribution characteristics of gas explosion accidents (Yi et al. 2017). Saleh made a claim for defense in depth as a guiding principle for safety in the mining industry, and pointed out the possible benefits to mining safety of adopting this hazard-centric structured systems approach (Saleh and Cummings 2011). Duzgun proposed a risk and decision analysis method for roof accidents (Duzgun and Einstein 2004). Wang conducted risk assessment of accident factors based on FAHP and developed a new safety management model (Wang et al. 2016). Liu established the China Mine Human Factors Analysis and Classification System (HFACS-CM) based on the statistical results of coal mine accidents, and combined with the Analytic Hierarchy Process (AHP) to systematically study the poor safety of coal miners and their related influencing factors (Liu et al. 2018). Sun conducted an investigation and research on coal mine water inrush accidents (Sun et al. 2016). Zhang conducted statistical analysis on particularly major accidents and put forward preventive countermeasures (Zhang et al. 2020). However, most of the work done by previous scholars mainly focused on a certain region or a certain type of coal accidents for statistical analysis, with a single angle of analysis and lack of unified regularity.

In order to summarize the occurrence rules of coal mine accidents in China from multiple perspectives, the coal mine accidents over the past 5 years (2017–2021) were studied through the steps of theoretical analysis, data collection, generalization, summarizing the regularities and making suggestions. Specific research methods are as follows: (1) Status of coal resource storage and mining. China's coal mine resources information is mainly obtained through the National Bureau of Statistics. Then, combined with the environment of coal mines in the new era, the safety production situation of China's coal mines is analyzed. (2) Coal mine accident data collection and analysis. The data from 2017 to 2021 is collected through the public information of the national and local coal safety bureaus, various government reports, relevant literatures and unpublished data of enterprises. Then, the data are classified from four aspects: accident level, accident type, accident area, and accident time. Finally, the accident statistics law is extracted from these data. (3) Make recommendations. Based on the results after statistical analysis, a new safety prevention model is established to suggest targeted prevention and control for coal safety in China.

## The basic situation of coal resources in China

### Coal resource storage characteristics

**Fig. 1:**
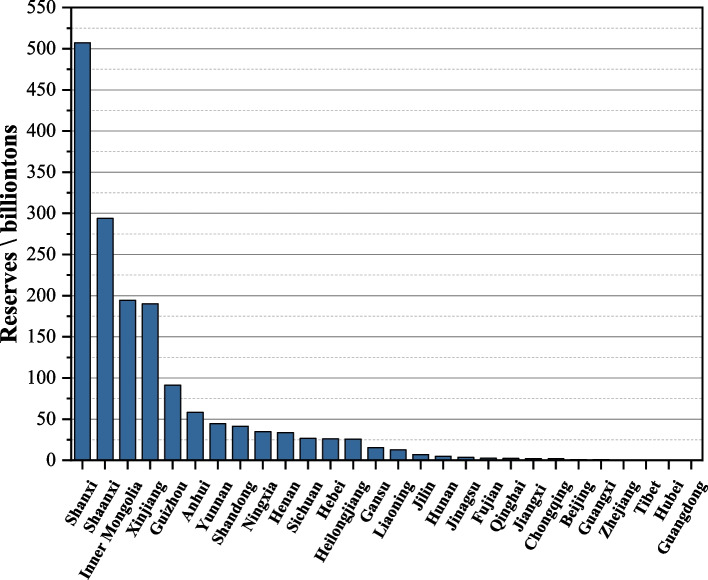
Distribution of coal reserves by province and city

Coal is the most abundant fossil energy source in the world, and about 80 countries in the world have coal resources (Zhang et al. 2021c). China is not only the largest coal producer in the world, but also the largest coal consumer and importer (Jia et al. 2021). However, the coal reserves suitable for surface mining in China are relatively small. According to statistics, China ranks fourth in the world in terms of coal reserves, with national coal reserves of about 162,288 million tons by the end of 2020 (Ministry of Natural Resources 2021). The general pattern of the geographical distribution of coal resources is more in the west and less in the east, rich in the north and poor in the south. The coal resources are mainly concentrated in Xinjiang, Inner Mongolia, Shanxi, Shaanxi, Guizhou, Ningxia and other regions, and are inversely distributed with the economic development of the region (Cao et al. 2020; Tang et al. 2020; Shao et al. 2020). As shown in Fig. 1, Shanxi Province has as much as 50.725 billion tons of coal reserves, accounting for 31.26% of the national reserves, ranking first in China; followed by Shaanxi and Inner Mongolia, with coal reserves of 29.390 billion tons and 19.447 billion tons respectively. Guangdong Province has the least amount of coal reserves, with only 0.01 billion tons. The western region is relatively rich in coal resources and generally of high quality, compared to the economically developed regions of China, which have a higher industrial output but a relative lack of coal resources. The coal resources in the developed eastern regions not only account for a smaller proportion, but also are more difficult to develop, having a more complex mining environment, higher mining costs and relatively poorer quality (Zhang et al. 2021a, b). Therefore, the limited economic investment in the development of resources in the west and the complex mining environment in the east are important reasons for the continuous occurrence of safety accidents during coal production.

### Coal resources mining characteristics

**Fig. 2:**
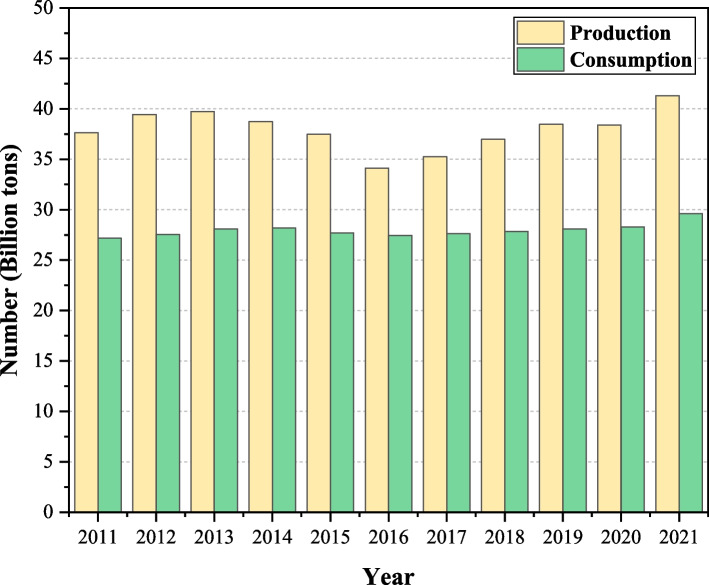
Changes in China's coal resources from 2011 to 2021

With the active promotion of China's carbon peaking and carbon neutralization work, China's energy supply has achieved remarkable results and production has grown steadily (Zhang et al 2022; Li 2021). As shown in Fig. 2, the raw coal production of China was 4.13 billion tons in 2021, up 5.7% year-on-year, and coal consumption was 2.96 billion tons, up 4.6% from the previous year (National Bureau of Statistics 2022). From the graph, we can also see that, with 2016 as the cut-off point, coal production decreases year by year in 2011–2015. While in 2016–2021, production gradually increases. This situation may be due to the tight supply of coal and the sharp increase in prices after 2016. Coal companies have made effort to increase production and supply and raw coal production has continued to grow. However, from the perspective of coal consumption, it has been in a stable state. Under the goal of "Dual Carbon", the growth of coal consumption is strictly controlled. By the end of 2021, coal consumption accounted for 56.0% of total energy consumption, with a decrease of 0.9 percentage points from the previous year. From 2011 to 2021, the proportion of coal in China's primary energy consumption structure decreases from 70.2% to 56.0%, but is still more than half. Coal-rich, oil-poor, and gas-poor resource characteristics in China determine that coal is the mainstay of China's energy consumption (Ning et al. 2014). With the long-term advancement of China's industrialization and urbanization, coal consumption will maintain a steady growth, but the transformation of economic growth mode and the implementation of energy conservation and emission reduction policies will slow down the growth rate of energy consumption.

**Fig. 3:**
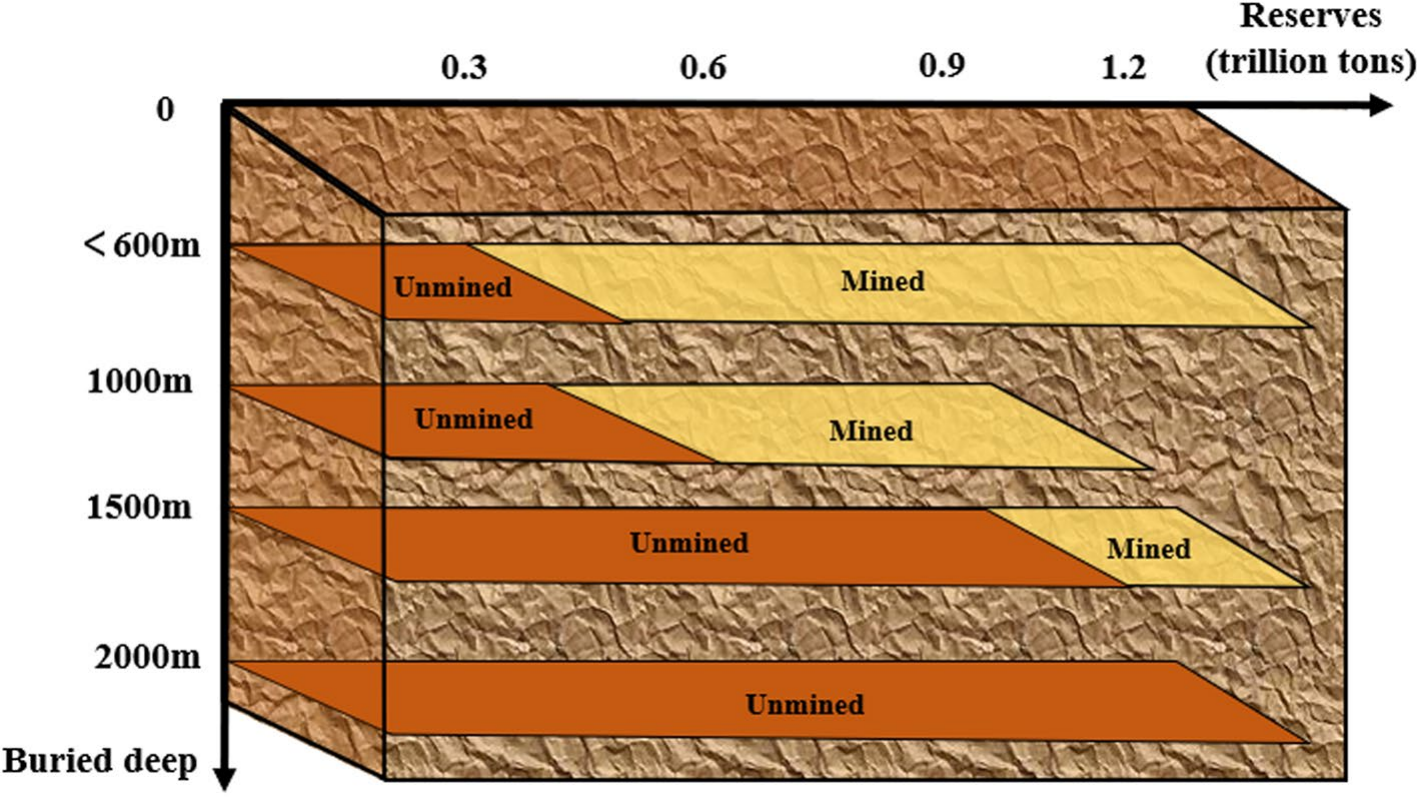
Coal resource storage and mining situation at different depths

In recent years, the social demand for coal resources has shown an obvious rising trend (Zhang et al. 2021a, b ), and the shallow coal resources cannot meet the needs of social development, so the depth of mining must be increased. According to statistics, the mining depth in China is increasing year by year, and the increase rate reaches about 10–25 m per year. As shown in Fig. 3, there are 47 mines with mining depth above 1000 m and the maximum mining depth reaches 1500 m (Zhao et al. 2020). At the same time, deep mining is becoming more and more difficult, and a lot of protective measures have been taken in mining safety every year, but the casualties are still very serious.

### Safety production situation in China's coal mine

In recent years, with the continuous improvement of coal mine safety laws and regulations, the level of safety technology and equipment has significantly upgraded, and the investment in safety production has increased significantly. In general, the safety production situation in China's coal mine has gradually improved, and the number of accidental coal mine deaths has continued to decline (Zeng and Ren 2019; Zheng et al. 2009; Chen 2020).

**Fig. 4:**
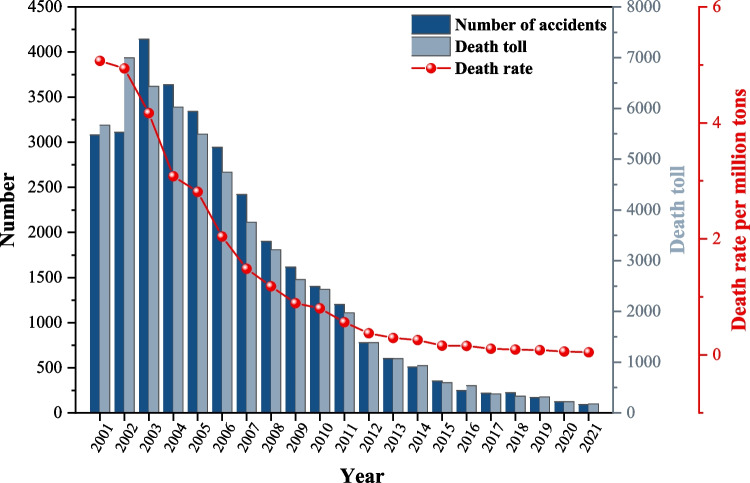
Statistics of coal mine accidents from 2001 to 2021

The last 20 years belong to the period of rapid improvement in the level of safety production. As can be seen from Fig. 4, the history of coal mine safety development is divided into two periods with 2017 as the node. Before 2017, the number of coal accidents in China peaked at 4143 in 2003 and then dropped significantly to 219 in 2017. As the government has paid close attention to coal safety in recent years and taken a series of safety measures, the death toll in coal mines dropped from 6995 in 2002 to 375 in 2017, with a decrease of 94.6%. At the same time, the death rate per million tons of coal mine declined in a more obvious trend, from 5.07 in 2001 to 0.106 in 2017, and coal mine safety has been effectively guaranteed.

After 2017, the pattern of coal mine safety showed a continuous and stable improvement. The death rate per million tons of coal mine exceeded the mark of 0.1 and has dropped to 0.044 by 2021, 58.5% lower compared to 2017. The number of accidents and deaths during 2017–2021 also showed a steady downward trend, reducing 128 cases and 197 people respectively. China is in the "13th Five-Year Plan" period over the past 5 years, it is an important opportunity period for the coal industry to accelerate the transformation and development and achieve the historical leap from big to strong. In the face of the complex macro environment, the difficult and heavy task of reform and development, especially the serious impact of the COVID-19, the whole industry implements to carry out the new national security energy strategy. Focus on intelligent mining and efficient intensive use of coal, focus on promoting scientific and technological innovation, focus on promoting industrial transformation and upgrading, China is entering a new chapter of high-quality coal development (Wang et al. 2018a, b). During the new "14th Five-Year Plan" period, it will be the most critical 5 years for the transformation and development of the coal industry. Therefore, the author starts from the accident statistics of 2017–2021, and analyzes the change of accident development law, which is of great significance to the prevention and control of coal mine accidents in China and even in the world.

## Statistical law of coal mine accidents in China

### Coal mine accidents level analysis

According to the Chinese Regulations on the Reporting and Investigation of Coal Mine Production Safety Accidents, coal mine accidents are classified into four classes based on the number of fatalities: general accidents (less than 3 people), major accidents (more than 3 and less than 10 people), catastrophic accidents (more than 10 and less than 30 people), and heavy catastrophic accidents (more than 30 people) (National Mine Safety Administration 2008).As shown in Table 1, the number of accidents and deaths of general accidents is the largest in different levels of coal mine accidents from 2017 to 2021, namely 692 and 783, accounting for 87.6% and 54.64% respectively. The number of major accidents and catastrophic accidents is 80 and 18 respectively, which is relatively small. No heavy catastrophic accidents have occurred from 2017 to 2021 and no deaths have resulted from this accident level.

**Table 1:** Number of accidents in different levels from 2017 to 2021

Year	General accidents	Major accidents	Catastrophic accidents	Heavy catastrophic accidents
2017	196	17	6	0
2018	205	17	2	0
2019	105	24	5	0
2020	109	10	3	0
2021	77	12	2	0

**Fig. 5:**
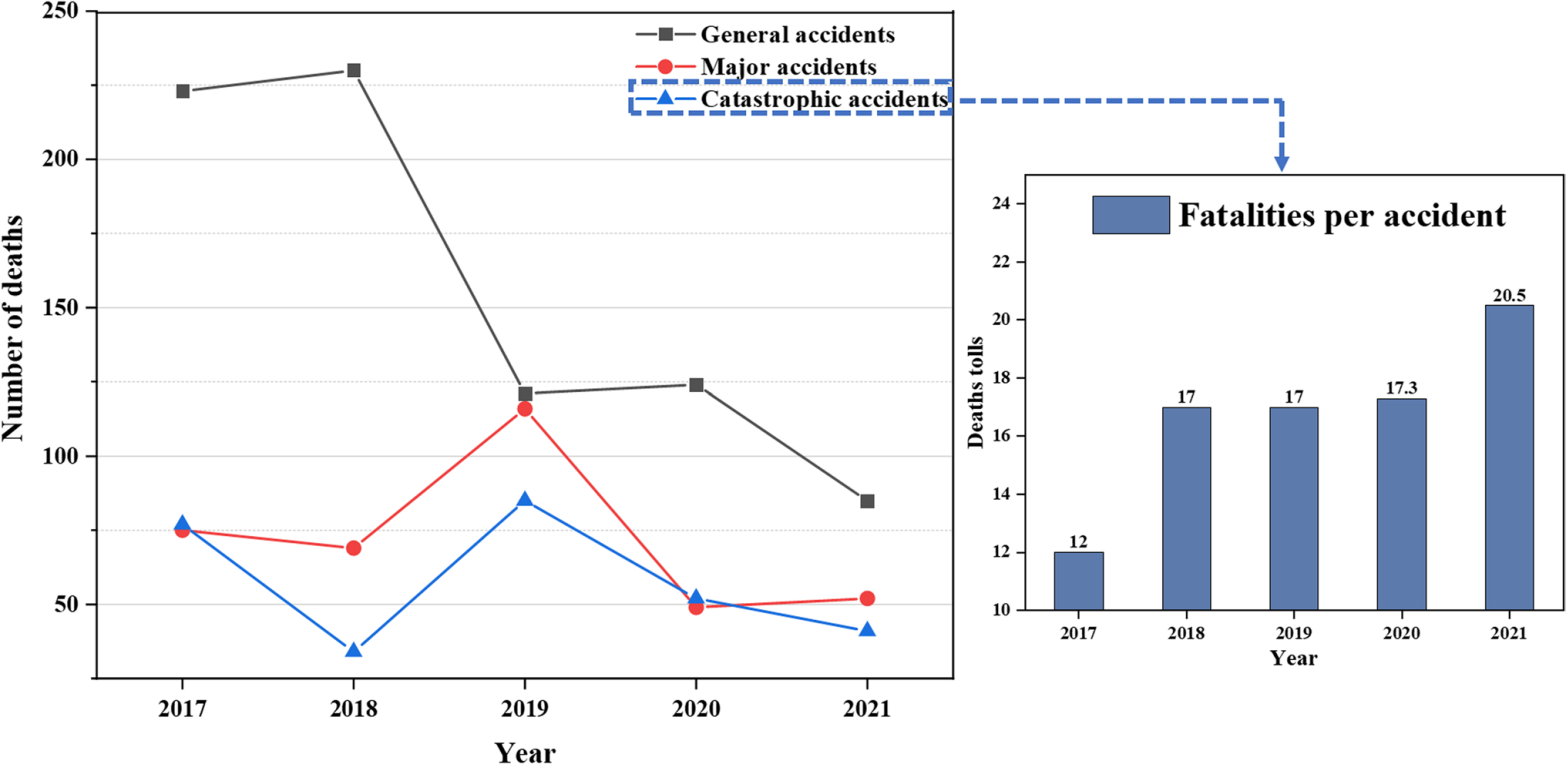
Number of deaths in different accidents level and fatalities per accident in catastrophic accidents

As can be seen from Table 1, the number of accidents at all levels showed an overall decreasing trend, and the number of major and catastrophic accidents was much smaller than that of general accidents. As can be seen from Fig. 5, the number of deaths in general accidents decreases year by year, while major and catastrophic accidents show a fluctuating decline. In particular, the number of fatalities in general accidents jumped down during 2018–2020, but the number of fatalities in the other two classes of accidents increased and then decreased. It may be that the number of deaths in major and catastrophic accidents rose during this period due to inadequate safety control of major disaster risks. When the government noticed the phenomenon and took a series of measures, the number of deaths returned to normal levels. However, for catastrophic accidents, the fatalities per accident showed an overall increasing trend during 2017–2021, which shows that the emergency response to major disaster risks needs to be further improved.

### Coal mine accidents type analysis

**Fig. 6:**
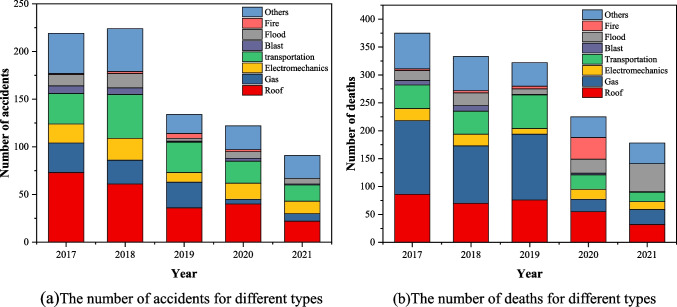
Number of accidents and death tolls based on different types

There are various types of coal mine accidents, and statistical analysis of different types of coal mine accidents can provide a basis for taking targeted prevention and control measures. According to the eight types of accidents in coal mines from 2017 to 2021, including gas accidents, roof accidents, flood accidents, fire accidents, electromechanics accidents, transportation accidents, blast accidents, and other accidents (Deng et al. 2014). As shown in Fig. 6, the number of roof, gas and transportation accidents have gradually decreased in 5 years. However, the decline in electromechanics and blast accidents is not obvious, and there is even a rising trend during 2017–2021. The above situation means that safety prevention and control in electromechanics and blast accidents need to be further improved. It is important to note flood and fire accidents, both with fluctuating development trends. This situation indicates that the relevant safety work is not in place and there is an urgent need to strengthen control, otherwise there will be devastating blows to coal mine safety in the future.

**Fig. 7:**
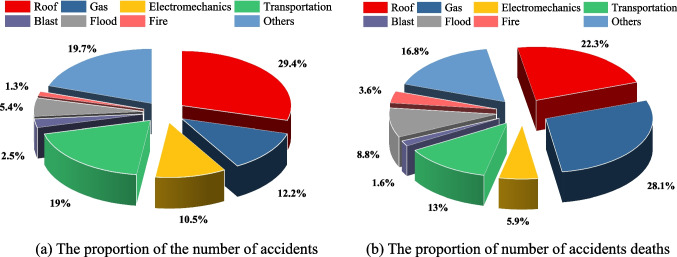
Proportion of quantity and death based on different types

Figure 7 shows the proportion of the total number of accidents and fatalities in each category. As shown in Fig. 7a, the number of roof accidents is the largest, accounting for 29.4%, followed by gas and transportation accidents, accounting for 19.7% and 19% respectively. The number of fire accidents occurs the least, only accounting for 1.3%. As shown in Fig. 7b, the number of deaths in gas accidents is the most, up to 28.1%, followed by roof and transportation accidents, accounting for 22.3% and 16.8%. However, blast accidents had the lowest number of deaths, only 1.6%. From the above analysis, it can be seen that the proportion of gas accidents during 2017–2021 is small, only 12.2%, while the proportion of death tolls in gas accidents is large. The main reason is that the mortality rate and damage of gas accidents is the most serious among all accident types. The risks posed by the gas accident mainly include gas outburst, gas explosion and associated dust hazards, etc. The risks associated with gas accidents mainly include gas deflagration, gas explosion and related dust hazards, which are more difficult to control.

### Coal mine accidents area analysis

**Fig. 8:**
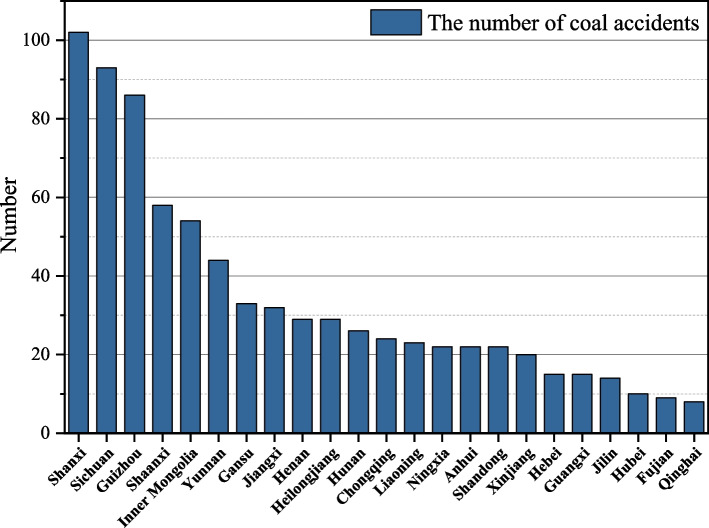
Distribution of the number of coal mine accidents in China

**Fig. 9:**
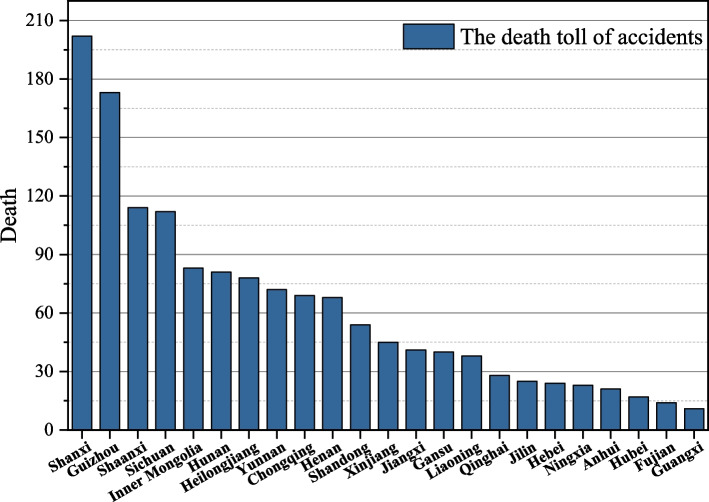
Distribution of the death tolls in coal mine accidents in China

China has complex topographic conditions and unstable coal seam inclination. At the same time, the distribution of coal mine accidents varies geographically due to the number of coal mines, production, geological conditions, mining status and other factors. Based on the distribution of coal mine accidents and deaths in different provinces, the data of coal mine accidents in each region are visualized, as shown in Figs. 8 and 9.

In terms of the number of accidents, Shanxi had the highest number of accidents, with a total of 102 coal mine accidents from 2017 to 2021, accounting for 12.9%. Followed by Sichuan and Guizhou, with 93 and 86 accidents respectively, accounting for 11.7% and 10.8%. The northern region of Inner Mongolia and Shaanxi also had more accidents, with 54 and 58 respectively. In terms of the number of deaths, Shanxi Province is still the most serious region, with 202 deaths, accounting for 14.1%, followed by Guizhou, Sichuan and Shaanxi. Although Shanxi Province ranks first in terms of the number of production safety accidents and the number of deaths, its average number of fatalities per accident is not high, only 1.98. Although only 24 coal mine accidents occurred in Chongqing during 2017–2021, the average number of fatalities was as high as 2.87.

Overall, China’s coal mine accidents in the geographical distribution have the law of "wide and more concentrated distribution, more in inland and less coastal, more in north and south and less in east and west". Most coal accidents are concentrated in Shanxi, Guizhou, Sichuan, Shaanxi, etc. The reason for this pattern is that coal production are larger and geological conditions are very complex in accident-prone provinces. Especially for Shanxi, the region is a large coal province, the number of coal mines and coal storage are among the top of China. The number of accidents and deaths in the province under the same circumstances is relatively more. At the same time, the accident-prone provinces do not pay enough attention to production safety, the government's supervision is weak and safety management policies are not comprehensive.

### Coal mine accidents time analysis

**Fig. 10:**
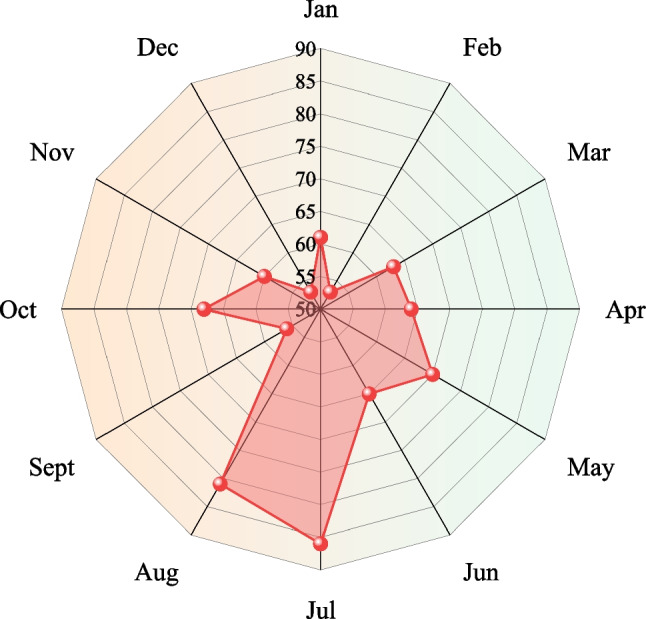
Number of accidents in different months from 2017 to 2021

**Fig. 11:**
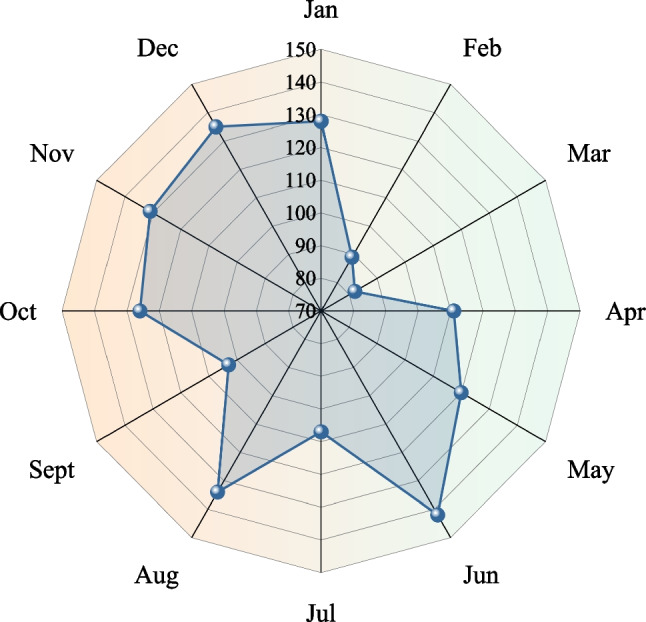
Death tolls in different months from 2017 to 2021

Through research and analysis, the frequency of coal mine safety accidents usually shows the concentration of time periods (Zhang et al. 2021a). Therefore, this study used the month as the time unit to form a period series and find the time point of accident frequency. Figures 10 and 11 show the statistics of the number of accidents and the number of fatalities in each month from 2017 to 2021.

In terms of the number of accidents, the high incidence of coal mine accidents is concentrated in July and August, with 86 and 81 accidents occurring respectively. Due to the hot weather in July and August, the surface air temperature and the humidity underground are high. Workers are prone to inattentiveness, fatigue, sleepiness and other physiological phenomena, resulting in slower reactions and less accurate movements. In addition, psychological phenomena such as emotional irritability and laziness are likely to occur because of the poor climatic environment, leading to violations. The number of accidents in February and December was relatively small, with only 53 coal mine accidents. The mines just started production and safety management was strengthened after the Spring Festival holiday in February. While the mines usually complete the final work in December and require the audit and inspect, so safety supervision is enhanced compared to the daily work period.

We can see from Fig. 11, the deaths in months showed a trend of "bipolar" distribution. There were fewer deaths in the first half of the year and more deaths in the second half. Specifically, the death tolls in June, August, November and December were more than other months. The number of deaths was the highest in June, up to 142 people, while the number of deaths in February and March were less, 89 and 82. This pattern is strongly related to weather changes throughout the year. The temperature is suitable in the spring and autumn, and the underground work environment is more comfortable. It’s easy to concentrate on the spirit and safety accidents can be well controlled. However, the weather is hot and dry in summer and is cold and wet in winter. If employees work for a long time in this environment, they will be exhausted in mind and body. Combined with regulatory negligence, many accidents will be happened frequently in the end.

**Fig. 12:**
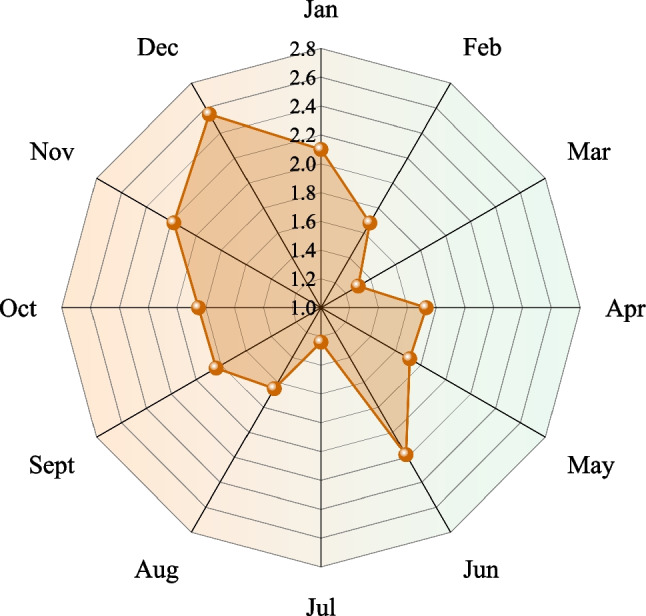
The average number of deaths in different months from 2017 to 2021

In order to more accurately describe the relationship between the number of accidents and deaths, we counted the average number of deaths per accident for different months from 2017 to 2021, as shown in Fig. 12. The average number of deaths was the highest in December with 2.55 per case. Compare with Figs. 10 and 11, it can be seen that although there were fewer accidents in December, the number of deaths was higher. This indicates that accidents in this month are generally more dangerous and deadly. The average number of deaths per accident in June and November was higher, namely 2.18 and 2.55 per case. However, the average number of deaths was the lowest in July, with 1.24 per case. The overall trend throughout the year shows a "wave" development in the average number of deaths. From the overall trend of the year, we can see that the average number of deaths shows a "wave-like" development, gradually decreasing from January to March, and gradually increasing from April to June, in a cyclical progression. This shows that the security prevention and control is not developed in a balanced way in time, and the management should not be relaxed in every month.

## Prevention measures

**Fig. 13:**
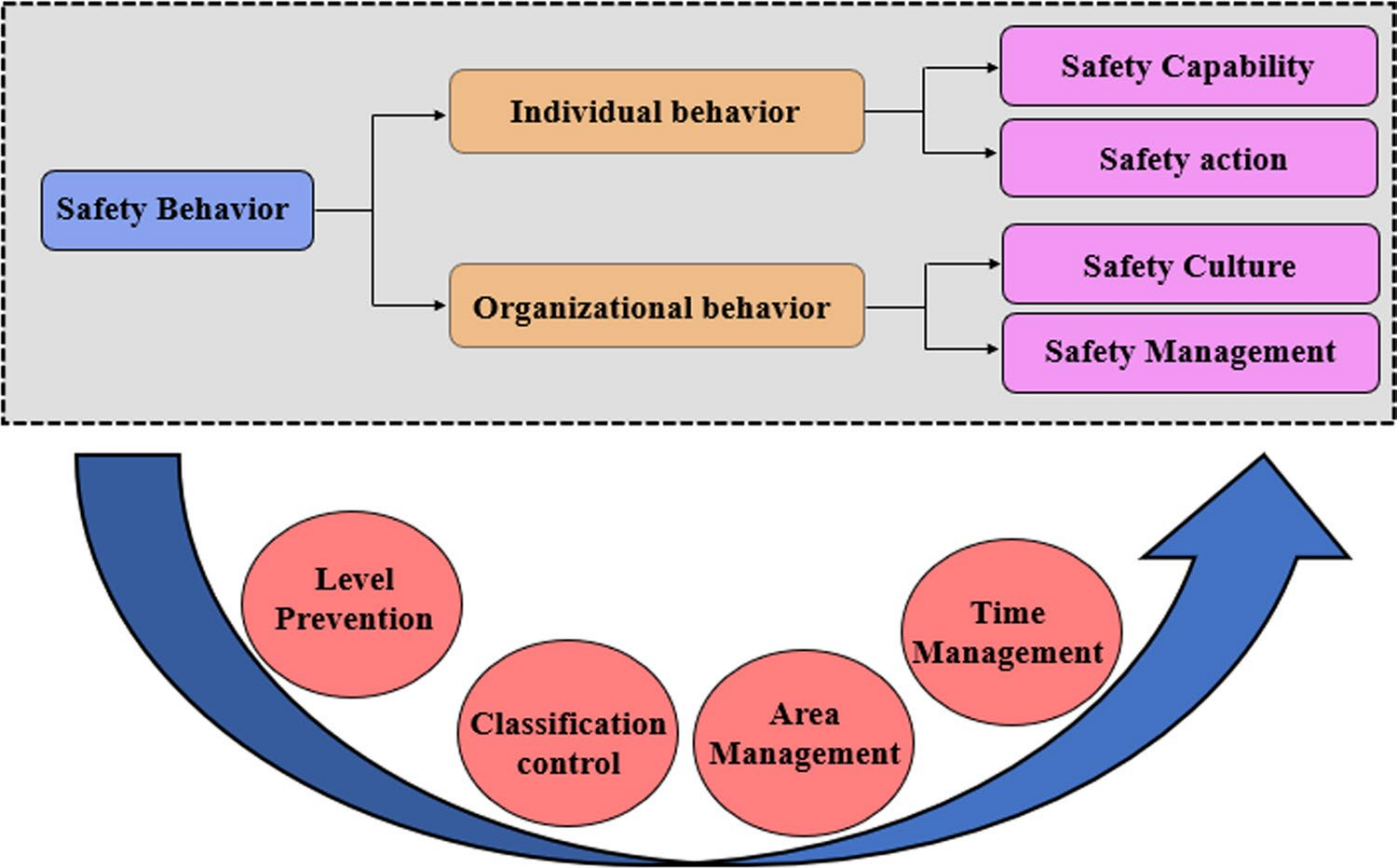
Classification system for behaviors in “4 + 4” model

There are many safety management systems in the world today, such as the ISO 45001 (Occupational Health and Safety Management Systems) (Liu et al. 2016). However, they are more complicated and are not targeted in mining industry (Soltanzadeh et al. 2022; Aryan et al. 2022; Siabi et al. 2022). In order to effectively prevent coal accidents and reduce casualties, the “4 + 4” safety management model is proposed based on accident statistics and situation of coal resources in China, as shown in Fig. 13. The model can effectively avoid accidents as well as casualties and systematically illustrate the management of safety behavior in coal mines, which can be targeted to prevent the development of coal mine accidents in four areas: “level”, “type”, “area” and “time”. The model covers a wide range of protection groups, from management leaders to junior employees. Besides, the model is not only applicable to coal mining enterprises, but also can be used by other companies with safety hazards. However, the “4 + 4” safety management model focuses on the influence of human behavior, ignoring the unsafe state of the machine and its influence. Further consideration will be given on how to incorporate the unsafe state of the machine into the model.

Firstly, safety behavior is divided into two categories: organizational behavior and individual behavior according to the accident process. On this basis, individual behavior is divided into two categories, namely safety capability and safety action, and organizational behavior is divided into two categories, namely safety culture and safety management. Finally, safety management is subdivided into four subcategories: level prevention, classification control, area management, and time management, by combining the latest statistical laws of coal accidents in China. In this way, the safety behavior is divided into “4 + 4” elements, i.e. "4 + 4" safety management model. Each of these elements is interrelated and works together to facilitate the completion of the safety behavior. Therefore, the authors propose specific countermeasures for safety prevention and control based on "4 + 4" safety model.

### Safety culture

Safety culture plays a guiding role in accident prevention, purely at the ideological level. However, safety culture is essential in accident prevention and control, especially in organizational factors (Wang et al. 2018a, b). In the past golden decade of coal mine development (2002–2011), excessive focus on economic benefits at the expense of employee life and health safety has led to frequent coal mine safety accidents. Since then, the government and various enterprises have introduced relevant policies and changed their management philosophy, gradually shifting from high economic income to low personnel fatalities, and the number of coal mine fatalities has rapidly decreased as a result. When there is a conflict between safety production and economic benefits, safety production must be placed before maximizing benefits. The next step is to change the traditional safety culture thinking. The content of the core concept of safety should evolve, which changes from the core concept of safety first and prevention-oriented safety to the development concept of life safety over everything. Relevant companies should also highlight the human-centered value and concern for life and health (Bu et al. 2021).

### Safety management

By reviewing the investigation reports of coal mine accidents, management defects are the main factors leading to the occurrence of safety accidents. Therefore, it is necessary to carry out in-depth publicity and education on coal mine production safety guidelines, policies, laws and regulations and lessons learned, and to urge and guide local governments and coal mine enterprises to strengthen safety management. We should improve the governmental supervision department, formulate flexible policies and regulations and increase the investment of resources. Combined with the previous accident statistics law, the safety management is subdivided into four subcategories of level prevention, classification control, area management, and time management, with the following specific recommendations.

1. Level prevention. The next step should focus on the safety prevention and control in general accidents. The government should increase investment in science and technology to promote intelligent mining. Enterprises need to further strengthen the safety responsibility system of enterprises and reduce the number of employees at dangerous work surfaces. In practice, employees should strictly abide by the operating specifications and prohibit single-person operations.
2. Classification control. Roof, gas, and transportation accidents have higher safety risks, so the government should invest more safety efforts in these types of accidents. For the roof accident, companies should adopt suitable mining methods and strictly require the technology of mining personnel and the design of the whole mining construction. Coal mining enterprises should also minimize the time of each mining and improve the speed of stratified mining as a way to protect the roof from damage. Employees should conduct frequent monitoring and inspection of roof safety. For gas accidents, government should establish a sound safety management system and pre-develop emergency plans for gas accidents. Small and medium-sized coal mining enterprises need to further improve the ventilation system to ensure that the ventilation capacity meets the needs of mine dilution and gas emission. Companies were advised to set up special gas accident teams to regularly analyze and forecast the disaster situation in the region, etc. For transportation accidents, each type of transportation equipment must be equipped with safety guards and be regularly inspected and maintained. Employees must be licensed to work and need to complete relevant transportation work under supervision, etc.
3. Area management. Coal mine safety investment should be increased in accident-prone provinces, especially in coal-producing regions such as Shanxi, Guizhou, and Shaanxi. Special coal mine safety investigation teams should be set up in each region to investigate and analyze the frequent occurrence of safety accidents in the region and update preventive measures in a timely manner. The supervision and management of small coal mines should be strengthened, and coal mines that do not comply with relevant regulations should be rectified and closed in a timely manner. Centralized control should be implemented to avoid management fragmentation. Government should also strengthen the supervision and management of small-scale coal mines, and promptly rectify and close those that do not comply with relevant regulations.
4. Time management. The number of underground staff should be reduced in summer and autumn. Companies should set reasonable working hours to avoid overworking employees. Safety managers are rotated on a regular basis to avoid accidental deaths due to management negligence during low accident periods. Relevant enterprises should develop a mining plan for each phase, and the work intensity should be averaged over time to avoid a sudden increase in workload during a certain period of time, etc.

### Safety capability

Government should increase investment in science and technology innovation and focus on building green and intelligent mines. The number of underground staff should be gradually reduced to promote the full mechanization of coal mines. The outdated production process and related equipment should be eliminated in time to improve the reliability of the equipment. Enterprises should strengthen the safety skills training of employees and regularly carry out safety education to improve the level of safety awareness of employees. Spiritual protection and material protection go hand in hand, allowing employees to participate in the supervision and management of safety.

### Safety action

Enterprises are advised to optimize task arrangements and eliminate fatigue operations. Companies should also treat employees fairly in terms of task allocation and bonuses to enhance employee trust. Operational specifications or work experience should be corrected in a timely manner to ensure that every operation of employees is safe and feasible. Coal mining companies need to conduct regular skills training and emergency drills to avoid accidents caused by improper operation.

## Conclusions

1. China's coal resources are geographically distributed in a pattern of more in the west and less in the east, rich in the north and poor in the south, and the distribution is inverse to the economic development. At the same time, the areas with large coal storage, such as Shanxi, Shaanxi, Inner Mongolia, etc., are prone to coal safety accidents. Raw coal production fluctuated upward after 2016, and the growth rate of coal consumption slowed down under the influence of Dual Carbon target. Deep coal mining will be the future development trend, which also increases the difficulty of safety accident control. The situation of coal mine safety production has gradually improved in the past 20 years, and the number of accidental deaths in coal mines has continued to decline. Safety prevention and control work still faces serious challenges in the context of COVID-19.
2. By analyzing the national coal mine accidents from 2017 to 2021, it can be seen that: the number of general accidents and deaths are the highest, and there is no heavy catastrophic accident during 2017–2021. Roof, gas and transportation accidents occur more frequently, and gas accidents cause the highest death tolls. In terms of geographical distribution, there are "wide distribution, more concentrated distribution, more inland, less coastal, more in north and south, less in east and west". Most coal accidents are concentrated in Shanxi, Guizhou, Sichuan, Shaanxi and safety situation in Shanxi Province is the most serious. Coal mine accidents are mainly concentrated in July and August, with fewer accidents in February and December.
3. The "4 + 4" safety management model is proposed. It divides safety behaviors into four categories: safety culture, safety management, safety capability, and safety action. At the same time, safety management is divided into four parts: level prevention, classification control, area management, and time management. Based on the statistical laws and the current situation of mining, the prevention countermeasures for coal accidents from these eight perspectives are proposed. It’s applicable to all coal mine enterprises in China as well as the world coal mine safety prevention and control.
